# A Cinderella story: how the vacuolar proteases Pep4 and Prb1 do more than cleaning up the cell’s mass degradation processes

**DOI:** 10.15698/mic2018.10.650

**Published:** 2018-08-18

**Authors:** Winnie Kerstens, Patrick Van Dijck

**Affiliations:** 1 VIB-KU Leuven Center for Microbiology, Kasteelpark Arenberg 31, B-3001 Leuven, Belgium.; 2 Laboratory of Molecular Cell Biology, Institute of Botany and Microbiology, KU Leuven, Kasteelpark Arenberg 31, B-3001 Leuven, Belgium.

**Keywords:** Pep4, Prb1, cathepsin D, protease, programmed cell death, SAGA, prion, Saccharomyces cerevisiae

## Abstract

Recently, several research groups have assigned non-vacuolar functions to the well-known *Saccharomyces cerevisiae* vacuolar proteases Pep4 and Prb1, which are also known as proteinases A and B. These non-vacuolar activities seem to be autophagy-independent and stress-induced and suggest an unexplored but possibly prominent role for the proteases outside the vacuole. The functions range from the involvement in programmed cell death, to protection from hazardous protein forms and regulation of gene expression. We propose that a deeper understanding of these molecular processes will provide new insights that will be important for both fungal biology as well as studies in mammalian cells, as they might open up perspectives in the search for novel drug targets. To illustrate this, we summarize the recent literature on non-vacuolar Pep4 and Prb1 functions in *S. cerevisiae* and review the current data on the protein homologs in pathogenic fungi.

## AN INTRODUCTION TO THE VACUOLAR PROTEASES: CLEANING WORKERS IN THE VACUOLE TO PRESERVE PROTEIN HOMEOSTASIS

In the model yeast *S. cerevisiae*, vacuolar proteases are known for the mass degradation of excess and senescent long-lived proteins, to preserve protein homeostasis. Generally, proteins destined for turnover reach the vacuole through the process of endocytosis or autophagocytosis 1,2. The substrate range of vacuolar proteases is wide and rather non-specific, assuring that the bulk of proteins gets degraded and constituents can be reused. So far, seven vacuolar proteases have been characterized in *S. cerevisiae*. They consist of two endoproteases called proteinase A and proteinase B encoded by *PEP4* and *PRB1*, two carboxypeptidases Y and S encoded by the genes *PRC1* and *CPS1*, two aminopeptidases I and Y encoded by *APE1* and *APE3* and a dipeptidylaminopeptidase encoded by *DAP2*. The group of Brodsky has reported on an eighth vacuolar protease encoded by *PFF1*, although its protease activity still needs to be demonstrated experimentally 3. The cytosolic proteasome represents another main site of protein breakdown in the cell, presumed to degrade specific short-lived and damaged proteins.

**Figure 1:**
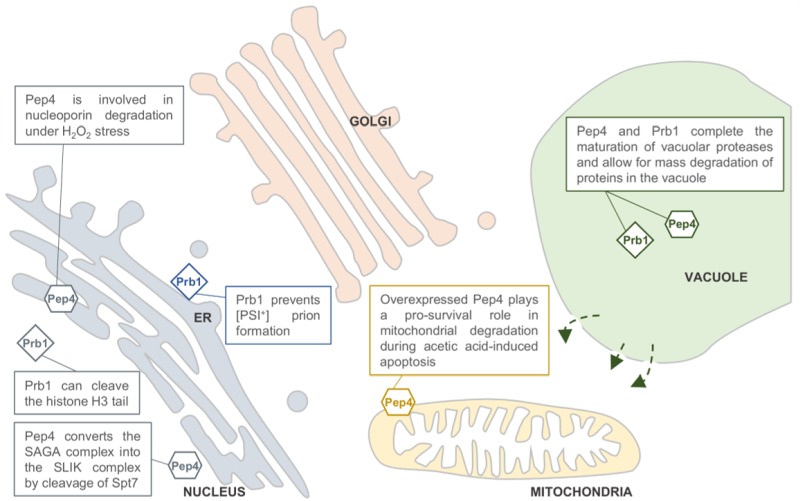
FIGURE 1: Predicted locations of the *Saccharomyces cerevisiae* proteases Pep4 and Prb1. Both proteases are well known for their role in protein degradation in the vacuole. Pep4 and Prb1 share a prominent role in the proteolytic activation and maturation of most vacuolar proteases, including themselves. Under stress conditions, the permeability of the vacuolar membrane has been reported to increase. This might enable the proteases to escape the vacuole and locate to other sites in the cell (indicated by arrows). It has been reported that Pep4 is associated with mitochondrial degradation under acetic acid-induced apoptosis. Furthermore, it has been described that nucleoporins are degraded by Pep4 in H_2_O_2_-induced cell death, suggesting a nucleus-associated localization. This idea is strengthened by the importance of Pep4 in the generation of the SAGA-like (SLIK) complex by cleavage of the Spt7 component of the SAGA complex. It was found that the conserved process of H3 clipping, altering gene expression, was performed by the Prb1 protease in *S. cerevisiae*, also suggesting a nuclear localization for Prb1. Furthermore, Prb1 was shown to inhibit [PSI^+^] prion formation. Okamoto *et al.* 6 hypothesize that prevention of prion formation by cleavage of the Sup35 protein takes place on actively translating ribosomes. It should be noted that the non-vacuolar localizations of Pep4 and Prb1 were never confirmed *in vivo* by conclusive experimental results.

Next to the degradative action of Pep4 and Prb1, the proteases also have a specific function inside the vacuole. With the exclusion of Dap2, all vacuolar proteases are synthesized as precursor proteins that become proteolytically activated and matured by the action of Pep4 and Prb1. This includes the precursors of Pep4 and Prb1 themselves. Pep4 stands at the top of this proteolytic activation cascade, as in *pep4* mutants the precursors of vacuolar proteases accumulate with little biochemical activity 4. Upon activation by Pep4, Prb1 is more often involved in the proteolytic processing of the precursor proteins, although Pep4 has redundant activity. A detailed review on the complex proteolytic activation cascade and transport routes of the vacuolar proteases can be found in 3,5 and references therein. Recently, studies on Pep4 and Prb1 activity also suggest non-vacuolar, specific substrates with diverse functions. In all these studies, the effect of the proteases was claimed to be autophagy-independent, often suggesting a non-vacuolar localization for Pep4 and Prb1. This is summarized in Figure 1.

## A CLOSER LOOK ON THE NON-VACUOLAR ACTIVITY OF PEP4 IN PROGRAMMED CELL DEATH

Pep4 shares 46% homology with human cathepsin D (CatD), a lysosomal protease 7. Similar to the human homolog, it has been reported that yeast Pep4 can be released from the vacuole under different kinds of stress, although the physiological relevance is unsure. Various studies propose that non-vacuolar Pep4 might play a role in stress-induced apoptosis. As such, Pep4 is responsible for the degradation of nucleoporins after H_2_O_2_ stress, an apoptotic process that is executed by caspases in mammalian cells 8. However, the functional significance of this action is unclear as in *S. cerevisiae* the degradation presumably takes place after the cells have lost their viability. Another study has indicated that Pep4 is released from the vacuole under acetic acid stress and plays a pro-survival role in mitochondrial degradation during acetic acid-induced apoptosis 9. A follow-up study confirmed that the protective role of Pep4 is dependent on its catalytic activity 10. According to Carmona-Gutierrez *et al*. 11, Pep4 carries anti-apoptotic and anti-necrotic activity during chronological aging. The anti-apoptotic activity depends on Pep4’s catalytic activity, while the anti-necrotic activity resides within the Pep4 precursor protein and is independent of its catalytic activity. It was shown that Pep4 overexpression resulted in a pro-survival effect during aging-induced cell death, that was caused by the anti-necrotic function only. This effect was accompanied by reduced histone H3 acetylation and dependent on increased spermidine biosynthesis. The interplay of histone acetylation levels and polyamine metabolism has been demonstrated by other labs as well, but the underlying molecular circuitry and the relevance to survival remain largely unidentified 12. A pro-survival signal of Pep4 was not detected under oxidative stress. Although overexpression of *PEP4* led to increased removal of oxidized proteins, it did not enhance lifespan 13. Altogether, these results show that vacuolar proteases affect programmed cell death in a stress-dependent manner. These relatively recent findings in yeast and the homology to mammalian processes opens perspectives for the clarification of the underlying molecular pathways. Ideally, this might lead to the identification of new drug targets, as various human diseases are caused by apoptosis-based mechanisms 9,14.

## PEP4 AND PRB1 INFLUENCE GENE EXPRESSION BY THE SPECIFIC CLEAVAGE OF NON-VACUOLAR SUBSTRATES

SAGA (Spt-Ada-Gcn5 acetyltransferase) is a multi-subunit histone modifying complex that is conserved from yeast to mammals and is known for its role in regulating gene expression 15. Interestingly, Pep4 can transform the SAGA complex into the SALSA or SAGA-like (SLIK) complex, by cleavage of the Spt7 component, leading to loss of its Spt8-binding domain 16. The function of SLIK has not been identified so far, but the authors report on an increased resistance to rapamycin when SLIK is present. Rapamycin is the inhibitor of the TORC1 complex, which regulates growth in response to nutrients and cellular stresses, suggesting a role for SLIK in the response to nutrient starvation 16.

It could be argued that Pep4 can thus influence gene expression both in a stimulatory and an inhibitory manner. Opposite to the gene inactivating histone hypoacetylation that accompanies Pep4 overexpression 11, generation of the SLIK complex links Pep4 with histone acetylation activity and thus gene activation. As the SAGA and SLIK complexes share at least thirteen subunits, some functional overlap between both is expected. Indeed, it has been demonstrated that the SLIK complex is capable of histone acetylation, similar to the SAGA complex 17. It might be that the functional difference between both complexes can be ascribed to a difference in the set of genes that become activated by their histone acetyltransferase (HAT) activity. This could be dependent on the properties of the unique components that make up the SAGA or SLIK complexes. Although the conversion of SAGA into SLIK might also alter the affinity to histone acetylation, which was not documented to our knowledge, it is more likely that Pep4 steers gene expression towards the induction of a certain subset of genes, by formation of the SLIK complex. As such, it could be hypothesized that the contrasting effects on histone acetylation associated with Pep4 converge on the activation of specific subsets of genes, while others are repressed.

Histone modifications are a common form of epigenetic regulation. The N-terminal cleavage of histone H3 is also a conserved process from yeast to mammals, but the biological significance is often unknown. The timing of cleavage depends on the specific cell type. In *S. cerevisiae, *it was found that Prb1 possesses histone H3 cleavage activity *in vitro* and that endogenous activity is absent in a *prb1*Δ deletion mutant 18. Previously, histone H3 clipping had already been associated with stationary phase and sporulation, consistent with the activity pattern of Prb1 and further confirming the H3 clipping activity of the protease 19. Histone cleavage by Prb1 can be considered a posttranslational modification that affects gene expression. It is hypothesized to either clip off transcription inhibitory marks at the histone tail or mark those nucleosomes that are to be displaced, to allow induction of gene expression 19. How Prb1 might localize to the nucleus was not addressed, however based on sequence analysis, the authors suggest that Prb1 contains a nuclear localization signal 18. No additional data regarding function or localization can be obtained from the closest human orthologue of Prb1, which is proprotein convertase subtilisin/kexin type 9 or PCSK9 20. This protease is extensively studied because of its function in cholesterol metabolism but there does not seem to be any overlap in function with the yeast Prb1 protease.

## THE ACTIVITY OF PEP4 AND PRB1 ON HAZARDOUS PROTEIN FORMS

Recently, it was observed that Pep4 can provide cytoprotection from human α-synuclein (αSyn), in a yeast model for Parkinson’s disease. Overexpression of αSyn led to cytosolic acidification and cell death, which could be overcome by overexpression of Pep4. Moreover, overexpression decreased αSyn oligomers and aggregates. For this, Pep4 required functional calcineurin, but the molecular framework needs further elucidation 21. Similarly, it was shown that overexpression of CatD protects against αSyn toxicity in mammalian cells and in the nematode *C. elegans* 22. It is remarkable that in *C. elegans* not only CatD overexpression but also spermidine supplementation rescue αSyn-induced pathology, albeit presumably in an autophagy-dependent manner 23. It is unclear whether CatD overexpression and spermidine supplementation converge on a similar response to αSyn toxicity as was demonstrated with aging-induced cell death in yeast, where the anti-necrotic effect of overexpressed Pep4 depended on spermidine biosynthesis 11.

Prions are infectious transmissible proteins that are self-propagating, turning healthy proteins into their prion isoforms. They cause diseases like mad cow disease in cattle and Creutzfeldt-Jakob disease in humans. In *S. cerevisiae*, multiple prions have been described 24. [PSI^+^] is the prion form of Sup35, also known as translation termination factor eRF3. The [PSI^+^] prion leads to repression of many stress-response genes 25. Interestingly, it was found that Prb1 can inhibit *de novo* [PSI^+^] prion formation by cleaving off a region of Sup35 N-terminally, which is crucial for the initial formation of aggregates 6. However, the protease cannot degrade existing [PSI^+^] prions. In all, this suggests that Prb1 can protect a cell from *de novo* [PSI^+^] prion formation. In this way it can enhance the general stress response of the cell. Although prions usually form amyloid structures, Roberts and Wickner 26 propose to consider any protein that can modulate itself *in trans* and be transmitted from one individual to another as a prion, without the need for amyloid formation or loss of function. Following this hypothesis, they demonstrated that active Prb1 is a prion, referred to as [β] that changes the conformation of its own precursor protein to mature Prb1.

## PEP4 AND PRB1 HOMOLOGS IN PATHOGENIC FUNGI: INTERESTING CANDIDATES TO TARGET IN THE COMBAT AGAINST FUNGAL INFECTIONS

Although the study of vacuolar proteases in pathogenic fungi is only beginning to develop, some studies already link their action to the pathogenicity of the species. For example, in the phytopathogenic species *Ustilago maydis*, the *pep4* gene was found to encode a vacuolar protease homologous to *Sc*Pep4. Deletion of this gene showed a strong effect on the yeast to mycelium dimorphic transition and reduced the virulence of the strain compared to the wild type strain 27. Also, in *Trichoderma* species, which are mycoparasitic fungi that are used as biocontrol agents of phytopathogenic fungi, secreted hydrolytic enzymes such as Prb1 are thought to play a key role in the process of mycoparasitism 28,29. The degree of homology between the mycoparasite Prb1 and yeast Prb1 is however unclear. In the human pathogen *Candida glabrata*, the protease homologs have only recently been identified, based on sequence homology. While their vacuolar localization was confirmed, additional functional studies are currently missing 30. In the *Candida albicans *genome database, three genes have been postulated as putative *ScPRB1* homologs, while the *Sc*Pep4 homolog has been characterized as the *Ca*Apr1 protein. Recent studies suggest a role for *Ca*Apr1 in morphological transition under nitrogen deficient conditions, and indicate a link between *CaAPR1* gene expression levels and the progression of Multiple Sclerosis disease in patients 31,32. This may be another example of how our microbiota may affect specific human diseases. In the human pathogen *Aspergillus fumigatus*, PEP2 was identified as a *Sc*Pep4 homolog and ALP2 was annotated as a *Sc*Prb1 homolog 33,34. Both proteins were found to be associated with the cell wall. Interestingly, Δ*alp2* mutants had a severe reduction in conidia formation and a slight growth defect. Moreover, ALP2 was found to be identical to a major allergen of *A. fumigatus*. These data support the idea that the proteases might be of importance in pathogenesis, and further research can confirm their medical relevance. This idea is reinforced by the recent identification of *A. fumigatus* PEP2 and ALP2 as potential targets for antifungal vaccines 35.

## CONCLUSION

This short overview illustrates the wide range of processes influenced by Pep4 and Prb1. It is evident that these proteases are not solely vacuolar degraders. Based on the preliminary reports in *S. cerevisiae*, the proteases seem to take part in a broad stress response mechanism, that is independent of autophagic processes and affects regulatory processes such as gene expression and programmed cell death. The molecular details however remain largely obscure. Further investigation of the non-vacuolar activities of Pep4 and Prb1 should provide the scientific community with an increased understanding of these general stress responses. It seems essential to determine the autophagy-dependency of Pep4 and Prb1 activities in future studies, to bring about a global understanding of the stress responses that are affected by these proteases. Current results in *S. cerevisiae* lead to the observation that autophagy-related stress responses rely solely on vacuolar Pep4 and Prb1 protease activity, while autophagy-independent actions of Pep4 and Prb1 occur outside of the vacuole. This idea should be validated further. Eventually, profound knowledge of these processes might identify specific participants as interesting new drug targets, both in fungal biology as well as in mammalian pathology.

## References

[1] Müller M, Schmidt O, Angelova M, Faseri K, Weys S, Kremser L, Pfaffenwimmer T, Dalik T, Kraft C, Trajanoski Z, Lindner H, Teis D (2015). The coordinated action of the MVB pathway and autophagy ensures cell survival during starvation.. Elife.

[2] Galluzzi L, Baehrecke EH, Ballabio A, Boya P, Bravo-San Pedro JM, Cecconi F, Choi AM, Chu CT, Codogno P, Colombo MI, Cuervo AM, Debnath J, Deretic V, Dikic I, Eskelinen EL, Fimia GM, Fulda S, Gewirtz DA, Green DR, Hansen M, Harper JW, Jaattela M, Johansen T, Juhasz G, Kimmelman AC, Kraft C, Ktistakis NT, Kumar S, Levine B, Lopez-Otin C (2017). Molecular definitions of autophagy and related processes.. EMBO J.

[3] Hecht KA, O'Donnell AF, Brodsky JL (2014). The proteolytic lanscape of the yeast vacuole.. Cell Logist.

[4] Woolford CA, Daniels LB, Park FJ, Jones EW, Van Arsdell JN, Innis MA (1986). The PEP4 gene encodes an aspartyl protease implicated in the posttranslational regulation of Saccharomyces cerevisiae vacuolar hydrolases.. Mol Cell Biol.

[5] Van Den Hazel HB, Kielland-Brandt MC, Winther JR (1996). Review: biosynthesis and function of yeast vacuolar proteases.. Yeast.

[6] Okamoto A, Hosoda N, Tanaka A, Newnam GP, Chernoff YO, Hoshino SI (2017). Proteolysis suppresses spontaneous prion generation in yeast.. J Biol Chem.

[7] Ammerer G, Hunter CP, Rothman JH, Saari GC, Valls LA, Stevens TH (1986). PEP4 gene of Saccharomyces cerevisiae encodes proteinase A, a vacuolar enzyme required for processing of vacuolar precursors.. Mol Cell Biol.

[8] Mason DA, Shulga N, Undavai S, Ferrando-May E, Rexach MF, Goldfarb DS (2005). Increased nuclear envelope permeability and Pep4p-dependent degradation of nucleoporins during hydrogen peroxide-induced cell death.. FEMS Yeast Res.

[9] Pereira C, Chaves S, Alves S, Salin B, Camougrand N, Manon S, Souisa MJ, Côrte-Real M (2010). Mitochondrial degradation in acetic acid-induced yeast apoptosis: the role of Pep4 and the ADP/ATP carrier.. Mol Microbiol.

[10] Pereira H, Azevedo F, Rego A, Sousa MJ, Chaves SR, Côrte-Real M (2013). The protective role of yeast cathepsin D in acetic acid-induced apoptosis depends on ANT (Aac2p) but not on the voltage-dependent channel (Por1p).. FEBS Lett.

[11] Carmona-Gutiérrez D, Bauer MA, Ring J, Knauer H, Eisenberg T, Büttner S, Ruckenstuhl C, Reisenbichler A, Magnes C, Rechberger GN, Birner-Gruenberger R, Jungwirth H, Fröhlich KU, Sinner F, Kroemer G, Madeo F (2011). The propeptide of yeast cathepsin D inhibits programmed necrosis.. Cell Death Dis.

[12] Hai Y, Shinsky SA, Porter NJ, Christianson DW (2017). Histone deacetylase 10 structure and molecular function as a polyamine deacetylase.. Nat Commun.

[13] Marques M, Mojzita D, Amorim MA, Almeida T, Hohmann S, Moradas-Ferreira P, Costa V (2006). The Pep4p vacuolar proteinase contributes to the turnover of oxidized proteins but PEP4 overexpression is not sufficient to increase chronological lifespan in Saccharomyces cerevisiae.. Microbiology.

[14] Aits S, Jäättelä M (2013). Lysosomal cell death at a glance.. J Cell Sci.

[15] Koutelou E, Hirsch CL, Dent SY (2010). Multiple faces of the SAGA complex.. Curr Opin Cell Biol.

[16] Spedale G, Mischerikow N, Heck AJ, Timmers HT, Pijnappel WW (2010). Identification of Pep4p as the protease responsible for formation of the SAGA-related SLIK protein complex.. J Biol Chem.

[17] Pray-Grant MG, Schieltz D, McMahon SJ, Wood JM, Kennedy EL, Cook RG, Workman JL, Yates JRr, Grant PA (2002). The novel SLIK histone acetyltransferase complex functions in the yeast retrograde response pathway.. Mol Cell Biol.

[18] Xue Y, Vashisht AA, Tan Y, Su T, Wohlschlegel JA (2014). PRB1 is required for clipping of the histone H3 N terminal tail in Saccharomyces cerevisiae.. PLoS One.

[19] Santos-Rosa H, Kirmizis A, Nelson C, Bartke T, Saksouk N, Cote J, Kouzarides T (2009). Histone H3 tail clipping regulates gene expression.. Nat Struct Mol Biol.

[20] Lin XL, Xiao LL, Tang ZH, Jiang ZS, Liu MH (2018). Role of PCSK9 in lipid metabolism and atherosclerosis.. Biomed Pharmacother.

[21] Aufschnaiter A, Habernig L, Kohler V, Diessl J, Carmona-Gutierrez D, Eisenberg T, Keller W, Büttner S (2017). The coordinated action of calcineurin and cathepsin D protects against α-synuclein toxicity.. Front Mol Neurosci.

[22] Qiao L, Hamamichi S, Caldwell KA, Caldwell GA, Yacoubian TA, Wilson S, Xie ZL, Speake LD, Parks R, Crabtree D, Liang Q, Crimmins S, Schneider L, Uchiyama Y, Iwatsubo T, Zhou Y, Peng L, Lu Y, Standaert DG, Walls KC, Shacka JJ, Roth KA, Zhang J (2008). Lysosomal enzyme cathepsin D protects against alpha-synuclein aggregation and toxicity.. Mol Brain.

[23] Büttner S, Broeskamp F, Sommer C, Markaki M, Habernig L, Alavian-Ghavanini A, Carmona-Gutierrez D, Eisenberg T, Michael E, Kroemer G, Tavernarakis N, Sigrist SJ, Madeo F (2014). Spermidine protects against α-synuclein neurotoxicity.. Cell Cycle.

[24] Liebman SW, Chernoff YO (2012). Prions in yeast.. Genetics.

[25] Baudin-Baillieu A, Legendre R, Kuchly C, Hatin I, Demais S, Mestdagh C, Gautheret D, Namy O (2014). Genome-wide translational changes induced by the prion [PSI+].. Cell Rep.

[26] Roberts BT, Wickner RB (2003). Heritable activity: a prion that propagates by covalent autoactivation.. Genes Dev.

[27] Soberanes-Gutiérrez CV, Juárez-Montiel M, Olguín-Rodríguez O, Hernández-Rodríguez C, Ruiz-Herrera J, Villa-Tanaca L (2015). The pep4 gene encoding proteinase A is involved in dimorphism and pathogenesis of Ustilago maydis.. Mol Plant Pathol.

[28] Geremia RA, Goldman GH, Jacobs D, Ardiles W, Vila SB, Van Montagu M, Herrera-Estrella A (1993). Molecular characterization of the proteinase-encoding gene, prb1, related to mycoparasitism by Trichoderma harzianum.. Mol Microbiol.

[29] Olmedo-Monfil V, Mendoza-Mendoza A, Gòmez I, Cortés C, Herrera-Estrella A (2002). Multiple envorinmental signals determine the transcriptional activation of the mycoparasitism related gene prb1 in Trichoderma atroviride.. Mol Genet Genomics.

[30] Sepúlveda-González ME, Parra-Ortega B, Betancourt-Cervantes Y, Hernández-Rodríguez C, Xicohtencati-Cortes J, Villa-Tanaca L (2016). Vacuolar proteases from Candida glabrata: acid aspartic protease PrA, neutral serine protease PrB and serine caroxypeptidase CpY. The nitrogen source influences their level of expression.. Rev Iberoam Micol.

[31] Amri Saroukolaei S, Ghabaee M, Shokri H, Khosravi A, Badiei A (2016). Evaluation of APR1 gene expression in Candida albicans strains isolated from patients with multiple sclerosis.. Jundishapur J Microbiol.

[32] Bauerová V, Hájek M, Pichová I, Hrušková-Heidingsfeldová O (2014). Intracellular aspartic proteinase Apr1p of Candida albicans is required for morphological transition under nitrogen-limited conditions but not for macrophage killing.. Folia Microbiol.

[33] Reichard U, Cole GT, Hill TW, Rüchel R, Monod M (2000). Molecular characterization and influence on fungal development of ALP2, a novel serine proteinase from Aspergillus fumigatus.. Int J Med Microbiol.

[34] Reichard U, Cole GT, Rüchel R, Monod M (2000). Molecular cloning and targeted deleton of PEP2 which encodes a novel aspartic proteinase from Aspergillus fumigatus.. Int J Med Microbiol.

[35] Ramirez-Garcia A, Pellon A, Buldain I, Antoran A, Arbizu-Delgado A, Guruceaga X, Rementeria A, Hernando FL (2018). Proteomics as a tool to identify new targets against Aspergillus and Scedosprorium in the context of cystic fibrosis.. Mycopathologia.

